# An Unusual Cause of Knee Pain Identified by Point-of-Care Ultrasound in the Emergency Department

**DOI:** 10.7759/cureus.86934

**Published:** 2025-06-28

**Authors:** Miguel F Agrait Gonzalez, Nehemesis Rivera Ortiz, Jenniffer Santiago, Glen Malaret Hernandez

**Affiliations:** 1 Emergency Medicine, Centro Médico Episcopal San Lucas, Ponce, PRI

**Keywords:** emergency medical service, morel-lavallee, msk ultrasound, point-of-care-ultrasound, soft tissue injury

## Abstract

Morel-Lavallée lesions (MLLs) are closed degloving injuries that typically result from shearing forces separating the subcutaneous tissue from the underlying fascia, creating a potential space that becomes filled with blood, lymphatic fluid, and necrotic tissue. These lesions are frequently missed on initial evaluation, especially in the context of blunt injury without obvious shearing trauma. Here, we present the case of a 28-year-old male who sustained trauma to his right thigh and knee while attempting to move a refrigerator. Initially diagnosed with a knee contusion after X-rays showed no fracture, the patient experienced worsening pain and swelling, prompting a visit to our emergency department (ED). Upon evaluation in the ED, the sonographic evaluation revealed a large fluid collection overlying the distal lateral thigh and lateral femoral condyle without any associated intraarticular effusion. Findings were consistent with an MLL, which was managed in the ED with ultrasound-guided drainage and compression therapy. This case highlights the importance of recognizing MLLs in the ED, as these injuries are frequently missed on initial evaluation. Early identification and appropriate management of MLL are crucial to preventing complications such as infection, chronic pain, loss of function, and delayed healing.

## Introduction

Morel-Lavallée lesions (MLLs) are a relatively rare type of closed soft tissue injury that most commonly results from a shearing force separating the subcutaneous fat from the underlying fascia. This then creates a potential space for fluid accumulation. The lesions were first described in the mid-19^th^ century and are typically associated with high-energy trauma, such as motor vehicle accidents or significant sports-related injuries [1]. MLLs most commonly occur over the greater trochanter of the femur, but they can also present in other areas, including the knee, thigh, and abdomen [2]. The condition can easily be misdiagnosed as a simple contusion or hematoma, especially in the early stages when the clinical presentation may appear mild or nonspecific [3]. Although most commonly described with shearing forces, it can also occur after blunt trauma without an obvious shearing component, as in this case.

Early and accurate diagnosis of MLLs is very important, as delayed recognition can lead to complications such as chronic fluid collection, infection, skin necrosis, chronic pain, and/or fibrosis. Historically, diagnosis has relied on advanced imaging modalities such as magnetic resonance imaging (MRI); however, this is not always feasible in the emergency department (ED). In recent years, point-of-care ultrasound (POCUS) has become a valuable diagnostic tool due to its accessibility, rapid results, and ability to differentiate fluid collections from other soft tissue, bone, or joint injuries in real time [4]. Despite its advantages, the application of POCUS for diagnosing MLLs in the emergency setting remains underreported, and its utility is not widely recognized in current practice guidelines. The diagnosis of MLL itself is also under-recognized in the general emergency medicine literature although it is well-recognized and described in the orthopedic and radiology literature. This overall lack of knowledge about the condition poses a significant risk to patients, as delayed diagnosis can lead to a host of complications or the need for more invasive surgical management down the line. The use of POCUS as a primary diagnostic tool in this context also allows for guidance of therapeutic interventions, such as needle aspiration or drainage [5]. By integrating ultrasound into the initial trauma evaluation, emergency physicians can expedite diagnosis and improve patient outcomes, potentially reducing the need for more invasive procedures in the future.

In this case report, we present a 28-year-old previously healthy male who sustained a direct blow to his right knee while moving a refrigerator. He denied any obvious shearing-type mechanism in this situation. He was initially evaluated at an outside facility on the day of the injury where X-rays of the right knee were performed and interpreted as normal. The patient was discharged with conservative management for presumed soft tissue injury; however, over the next three days, his right knee and distal thigh continued to swell, prompting him to seek further evaluation in our ED. POCUS evaluation revealed a large fluid collection consistent with an MLL over the distal lateral thigh/upper lateral knee, which was subsequently drained under ultrasound guidance. This case underscores the diagnostic value of POCUS in the ED and highlights the importance of considering MLLs as a possible diagnosis in patients presenting with an acute injury, particularly in the setting of a shearing-type mechanism [6].

## Case presentation

A 28-year-old previously healthy male presented to the ED with complaints of right knee pain and progressive swelling three days after a direct blow to the right knee. The injury occurred while the patient was moving a heavy refrigerator, and his knee became momentarily stuck between the wall and the refrigerator itself. He denied any shearing-type force and stated that he was able to move the refrigerator off his leg quickly. This event resulted in immediate pain and localized swelling. He was initially evaluated at an urgent care facility, where knee X-rays were performed and reported as normal. The patient was discharged with a diagnosis of a contusion and advised to use ice and over-the-counter analgesics.

Upon arrival at our ED, the patient reported worsening swelling and pain, particularly on the medial aspect of the right upper knee, limiting his ability to walk. He denied any history of fever, erythema, or systemic symptoms but noted increased discomfort with most right knee movements, in particular with deep knee flexion, which acutely worsened the pain. His vital signs were: temperature (T) 36.5°C, heart rate (HR) 88 beats per minute, blood pressure (BP) 132/79 mmHg, respiratory rate (RR) 16 breaths per minute, and oxygen saturation (SpO2) 97%.

**Figure 1:**
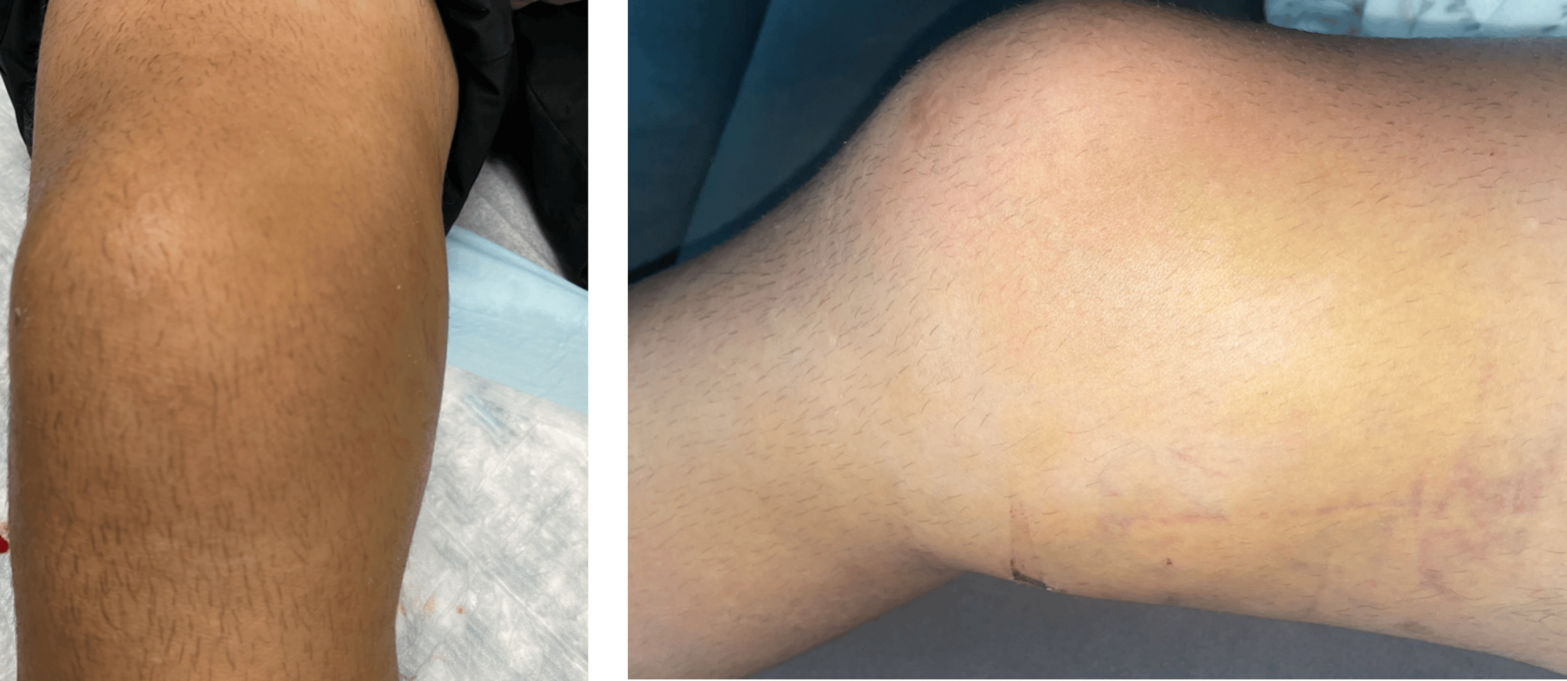
Gross image of the patient's affected (right) knee showing marked swelling and bruising on the medial aspect of the knee

On examination, there was obvious medial swelling of the right knee without any open wounds (Figure 1).

Range of motion was limited due to pain, affecting both passive and active flexion and extension. Palpation revealed significant tenderness over the superomedial aspect of the knee, along with a palpable, fluctuant fluid collection initially suggestive of an effusion, although both the patellar ballottement and bulge sign were negative. There was no tenderness across the joint line or over the quadriceps and patellar tendons. The neurovascular assessment was intact, demonstrating 5/5 strength (based on the Oxford Scale/Medical Research Council Manual Muscle Testing scale) in leg extension, plantar flexion, and dorsiflexion of the foot. Special tests, including the Lachman test, were normal, and there was no laxity or pain observed with valgus or varus stress testing.

**Figure 2:**
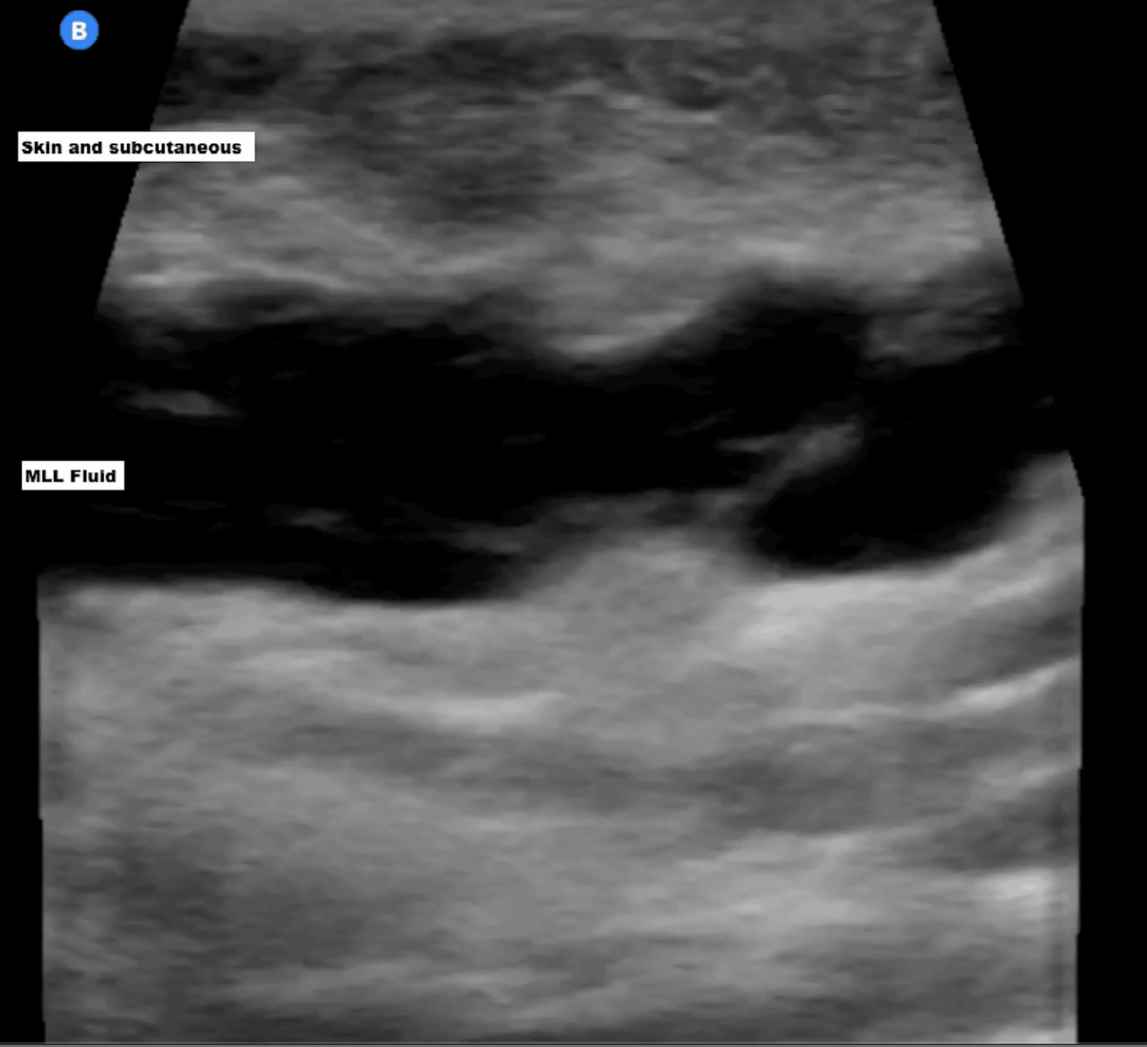
POCUS image of an MLL showing fluid collection between the subcutaneous tissue and muscle in the medial distal thigh MLL: Morel-Lavallée lesion; POCUS: point-of-care ultrasound

**Figure 3:**
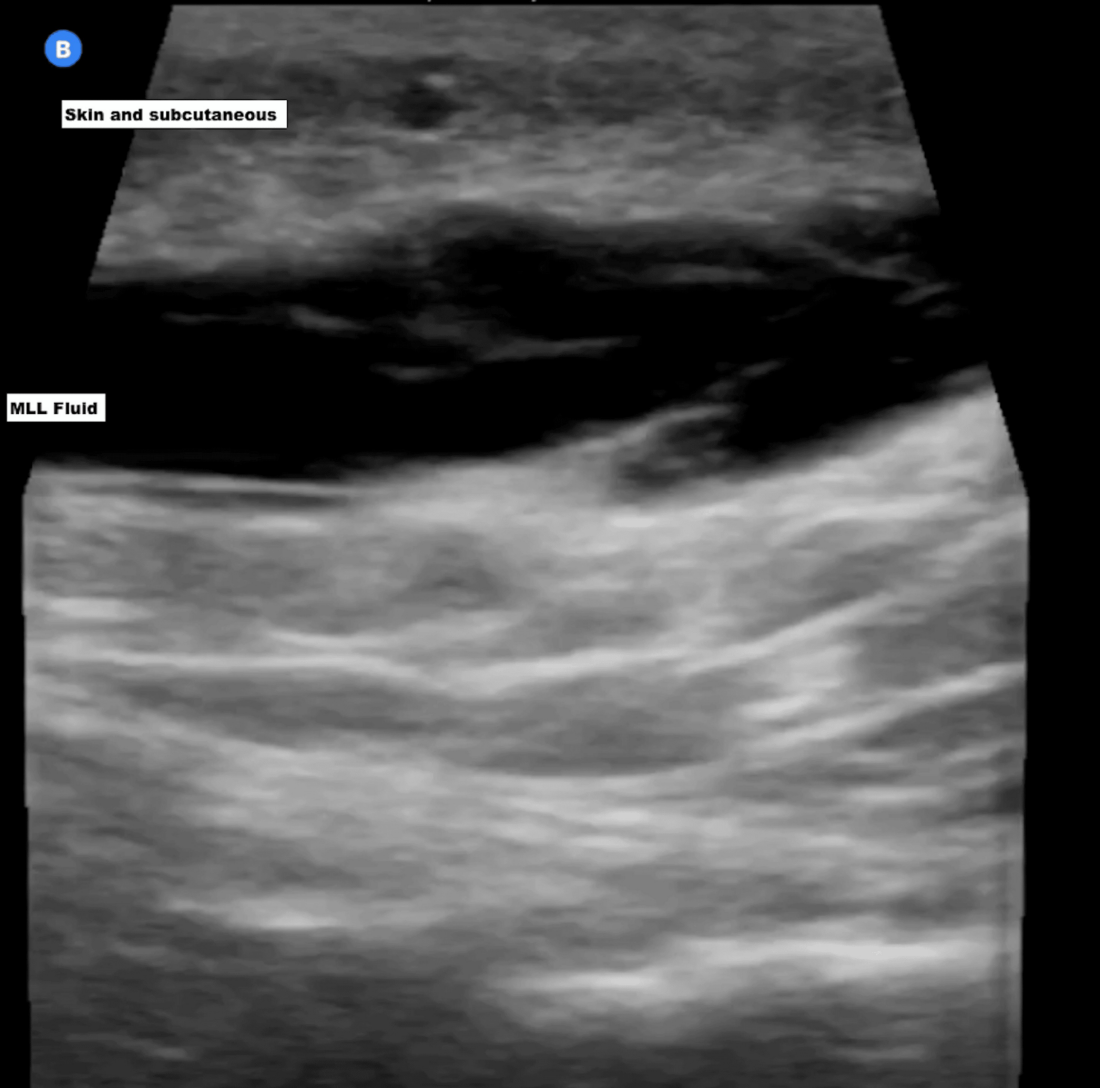
POCUS image of MLL in the right distal thigh and knee, showing fluid collection between the subcutaneous tissue and the muscle layer MLL: Morel-Lavallée lesion; POCUS: point-of-care ultrasound

Given the patient’s history of trauma and the concerning physical findings, POCUS was utilized for further evaluation. Ultrasound imaging revealed a large, hypoechoic fluid collection between the subcutaneous tissue and the fascial layer, consistent with an MLL (Figures 2, 3).

There was no evidence of a right knee joint effusion or other pathologic findings on ultrasound evaluation to suggest internal derangement or ligament injury of the right knee. 

**Figure 4:**
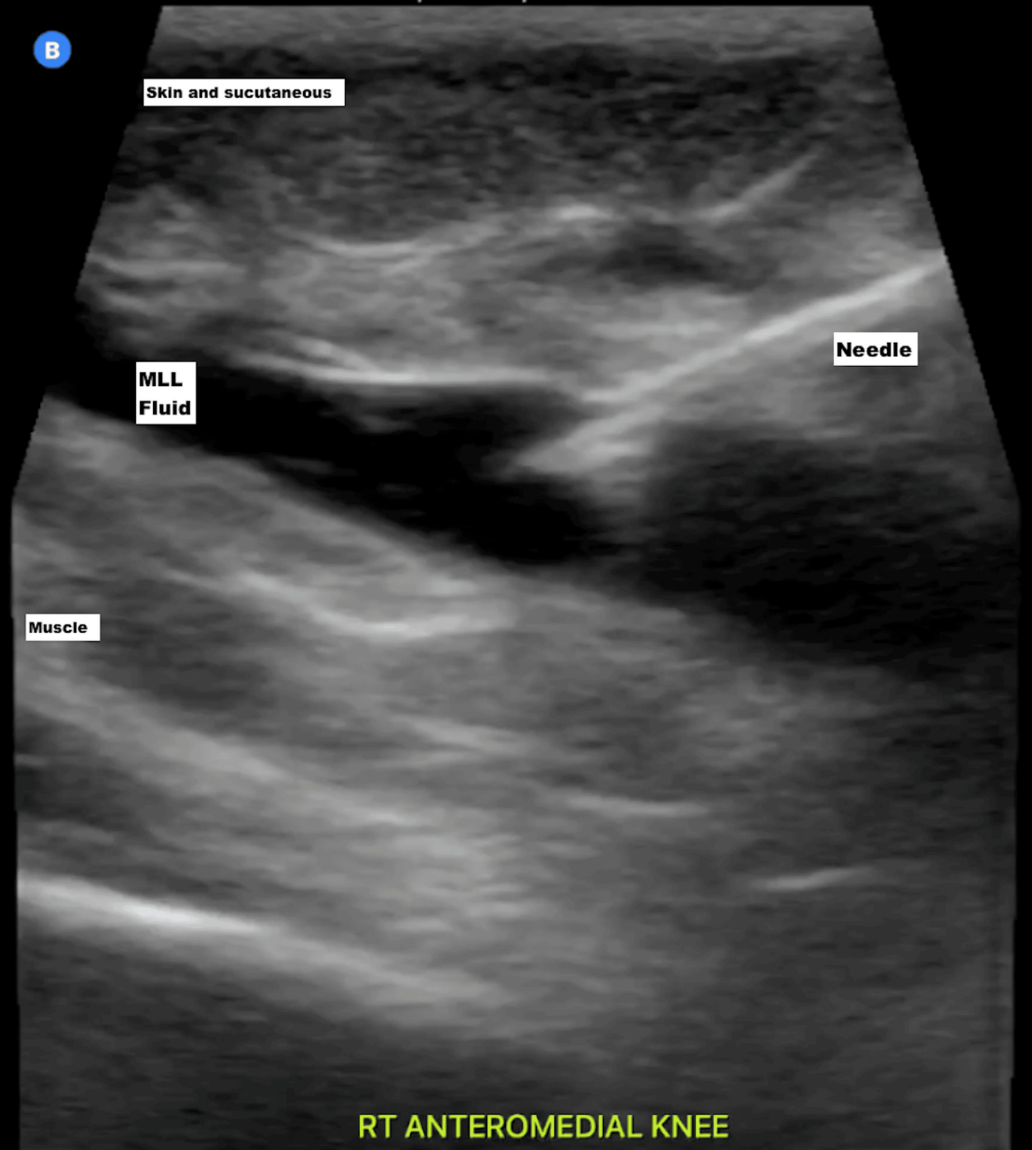
POCUS-guided needle drainage showing the needle visualized within the MLL fluid collection MLL: Morel-Lavallée lesion; POCUS: point-of-care ultrasound

Following the diagnosis of an MLL, the decision was made to proceed with drainage of the fluid collection in the ED. Using a sterile technique and strict ultrasound guidance (Figure 4) 

**Figure 5:**
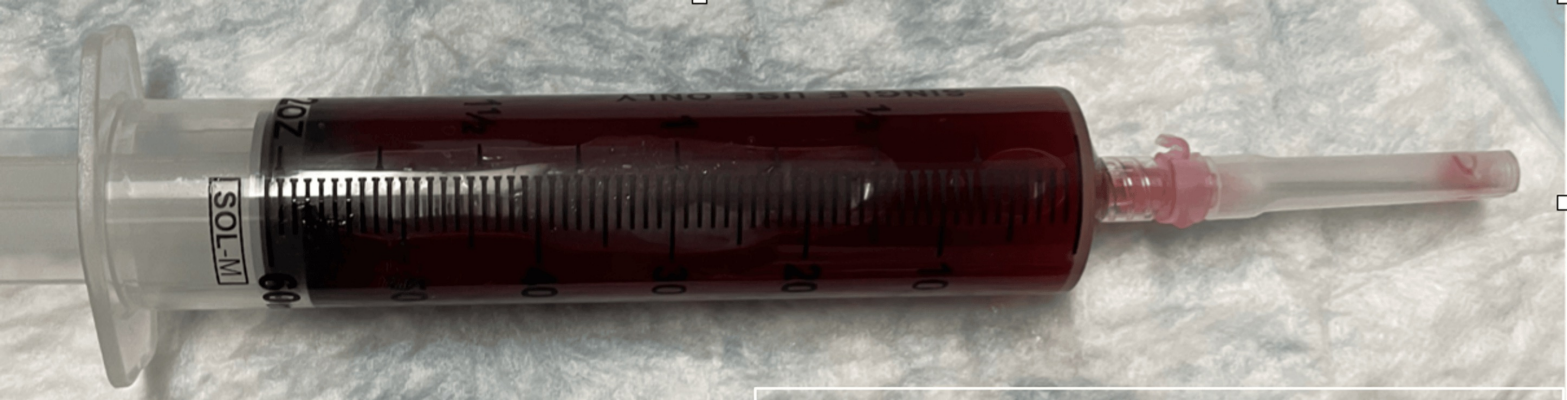
Aspirated MLL: the fluid, although bloody, is very thin and watery MLL: Morel-Lavallée lesion

Approximately 60 mL of thin, bloody, and watery fluid was aspirated from the lesion (Figure 5).

**Figure 6:**
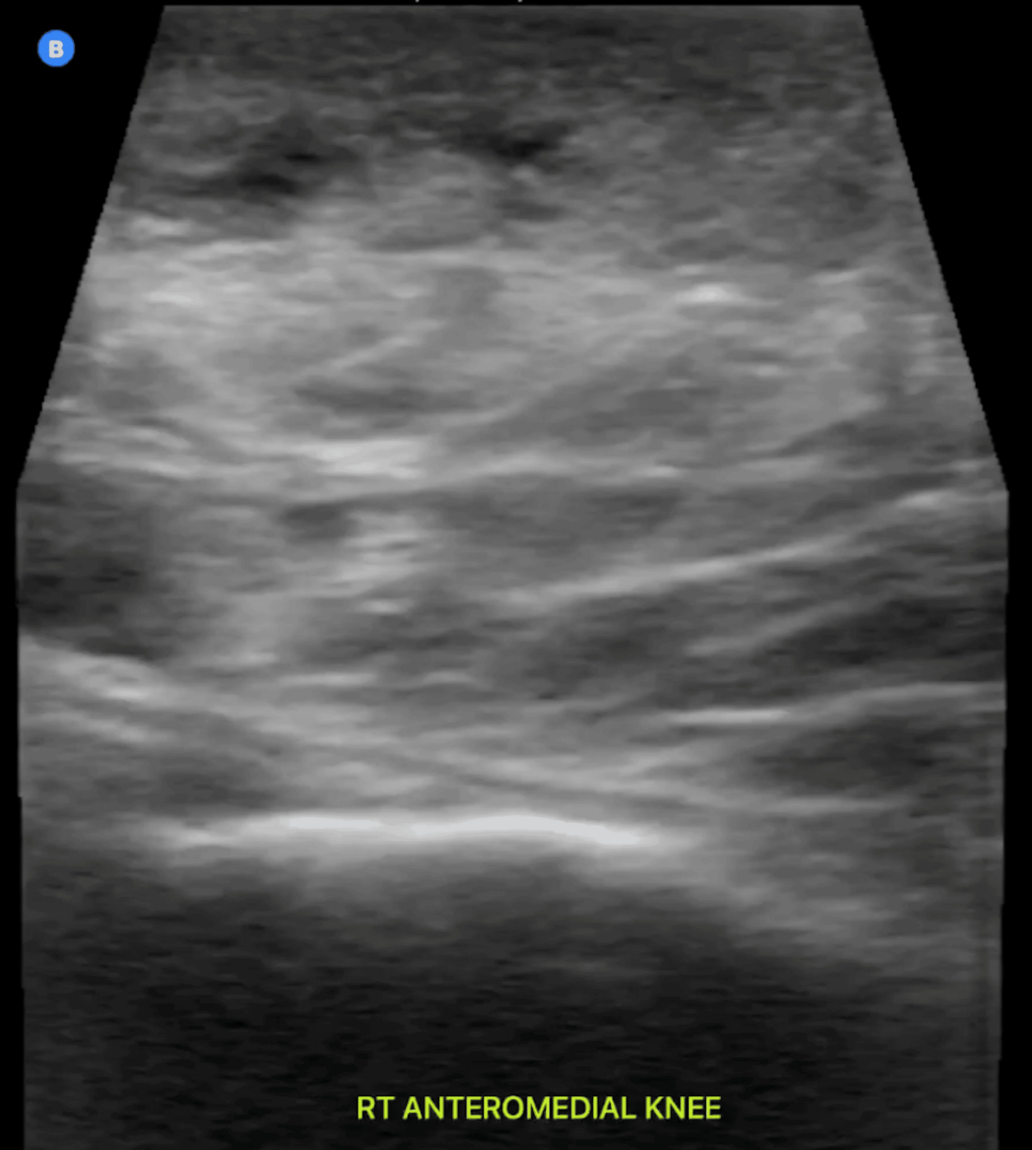
Post-drainage image of the MLL site showing complete resolution of the fluid collection MLL: Morel-Lavallée lesion

A compressive dressing was applied after the procedure, and the patient was instructed on continued use of compression at home. Post-drainage ultrasound showed complete resolution of MLL (Figure 6).

The patient experienced significant relief of symptoms following the drainage procedure and was ambulatory with only minimal discomfort in the ED. There was no suspicion of infection upon evaluation, so prophylactic antibiotics were not provided in this case. At a two-week follow-up evaluation in the outpatient sports medicine clinic, there was no evidence of recurrent fluid accumulation, and the swelling had resolved completely. The patient reported no further pain and was able to return to normal activities without the need for additional interventions, such as surgical debridement or sclerotherapy.

## Discussion

MLLs are uncommon but significant post-traumatic soft tissue injuries, characterized by closed degloving in which a shearing force separates the subcutaneous tissue from the underlying fascia [1,6]. This separation creates a potential space that can fill with blood, lymph, and necrotic fat, leading to a distinct fluid collection. While MLLs were initially described in the context of pelvic trauma, they are increasingly recognized in other locations, including the thigh, knee, and lower extremities, especially following blunt trauma, as seen in our case. The relative rarity and variable presentation of MLLs often lead to misdiagnosis or delayed diagnosis, which can result in complications such as chronic seroma formation or secondary infection [7].

In this case, the patient presented with a progressive swelling of the right knee following direct blunt trauma. The initial evaluation at an outside facility included X-rays, which were normal, leading to a diagnosis of a contusion. This underscores a common challenge in diagnosing MLLs: the clinical presentation may mimic other soft tissue injuries, and standard radiographs often fail to identify the underlying lesion. As demonstrated here, the use of POCUS in the ED was pivotal in correctly diagnosing the MLL [5,8]. Ultrasound findings of a hypoechoic fluid collection between the subcutaneous tissue and fascia, which is easily compressible, are highly suggestive of MLL, making it a valuable diagnostic tool, especially when physical examination findings are ambiguous [5].

The management of MLLs remains variable and is influenced by the size, chronicity, and location of the lesion. In acute cases, like the one presented, early recognition and intervention can prevent complications and reduce the need for surgical management [7]. Our decision to perform ultrasound-guided aspiration in the ED was based on the fluid collection and the patient’s symptomatic presentation. The aspiration of approximately 60 mL of thin, bloody fluid provided immediate symptomatic relief and was followed by the application of a compressive dressing, a strategy supported by the literature as an effective initial management approach [9,10].

Conservative management, including aspiration and compression, is often successful for acute or small MLLs. However, chronic or recurrent lesions may require more invasive approaches, such as surgical debridement, sclerotherapy, or even excision [11]. Our patient’s resolution without the need for additional interventions aligns with findings from previous studies suggesting that early drainage and compression therapy can be curative in select cases, particularly when performed promptly after injury. Compression therapy alone may be tried for smaller MLL, although drainage is generally preferred in addition to compression therapy to aid in complete resolution [8,10]. 

This case highlights the effective use of POCUS for the rapid diagnosis and management of an MLL in the emergency setting and demonstrates the successful management of the lesion with bedside aspiration and compression therapy, avoiding more invasive procedures. Additionally, this case highlights the utility of POCUS as an accessible and non-invasive diagnostic tool in the ED. Traditional imaging modalities such as MRI are considered the gold standard for MLL diagnosis due to their ability to delineate the extent of soft tissue damage and fluid composition. However, MRI is not always readily available in emergency settings, and delays in imaging may postpone appropriate management. Ultrasound, on the other hand, allows for rapid bedside assessment, aiding in early diagnosis and guiding interventions such as fluid aspiration. This aligns with current trends emphasizing the increasing role of POCUS in the evaluation of soft tissue injuries, particularly in emergency settings where timely decision-making is critical.

This case contributes to the growing body of literature supporting the use of ultrasound for both the diagnosis and management of MLLs in the acute setting. Given the variability in presentation and the potential for delayed or missed diagnoses, increased awareness of MLLs among emergency clinicians is crucial. Recognizing the typical clinical and ultrasound imaging findings can expedite appropriate intervention and prevent complications.

## Conclusions

This case underscores the importance of considering an MLL in patients presenting with persistent swelling following traumatic injury, even when initial radiographs are unremarkable. The use of POCUS enabled a prompt and accurate diagnosis, facilitating successful management with bedside aspiration and compression. Increased awareness and utilization of ultrasound in similar presentations may enhance diagnostic accuracy and improve patient outcomes, reducing the need for more invasive procedures.

Future research should focus on developing standardized protocols for the diagnosis and management of MLLs in the ED, as well as exploring the role of novel therapeutic interventions for chronic or recurrent cases. Comparative studies evaluating the outcomes of nonsurgical versus surgical management strategies or early versus delayed drainage, particularly in different clinical settings, would also be valuable in guiding best practices.
